# Contrastive virtual staining enhances deep learning‐based PDAC subtyping from H&E‐stained tissue cores

**DOI:** 10.1002/path.6491

**Published:** 2025-11-04

**Authors:** Maximilian Fischer, Alexander Muckenhuber, Robin Peretzke, Luay Farah, Constantin Ulrich, Sebastian Ziegler, Philipp Schader, Lorenz Feineis, Hanno Gao, Shuhan Xiao, Michael Götz, Marco Nolden, Katja Steiger, Jens T Sieveke, Lukas Endrös, Rickmer Braren, Jens Kleesiek, Peter Schüffler, Peter Neher, Klaus Maier‐Hein

**Affiliations:** ^1^ German Cancer Research Center (DKFZ) Heidelberg, Division of Medical Image Computing Heidelberg Germany; ^2^ Medical Faculty Heidelberg University Heidelberg Germany; ^3^ German Cancer Consortium (DKTK), DKFZ Core Center Heidelberg Heidelberg Germany; ^4^ Research Campus M^2^OLIE Mannheim Germany; ^5^ Institute of Pathology, TUM School of Medicine and Health Technical University of Munich Munich Germany; ^6^ Bridge Institute of Experimental Tumor Therapy (BIT), Division of Solid Tumor Translational Oncology (DKTK) and Department of Medical Oncology, West German Cancer Center, University Hospital Essen University of Duisburg‐Essen Essen Germany; ^7^ Helmholtz Imaging, DKFZ Heidelberg Germany; ^8^ Faculty of Mathematics and Computer Science Heidelberg University Heidelberg Germany; ^9^ Clinic of Diagnostics and Interventional Radiology, Section Experimental Radiology Ulm University Medical Centre Ulm Germany; ^10^ National Center for Tumor Diseases (NCT), NCT Heidelberg a partnership between DKFZ and University Medical Center Heidelberg Heidelberg Germany; ^11^ German Cancer Consortium (DKTK), partner site Munich, a partnership between DKFZ and School of Medicine and Health Technical University of Munich Munich Germany; ^12^ German Cancer Consortium (DKTK), partner site Essen, a partnership between German Cancer Research Center (DKFZ) and University Hospital Essen Essen Germany; ^13^ Institute for Diagnostic and Interventional Radiology, School of Medicine and Health, TUM University Hospital Technical University of Munich Munich Germany; ^14^ Department of Diagnostic and Interventional Radiology and Nuclear Medicine University Medical Center Hamburg‐Eppendorf Hamburg Germany; ^15^ Institute for AI in Medicine (IKIM) University Medicine Essen Essen Germany; ^16^ Munich Center for Machine Learning Munich Germany; ^17^ Pattern Analysis and Learning Group, Department of Radiation Oncology Heidelberg University Hospital Heidelberg Germany

**Keywords:** virtual staining, PDAC subtyping, contrastive learning, digital pathology, CycleGAN, H&E, IHC

## Abstract

Pancreatic ductal adenocarcinoma (PDAC) subtyping typically relies on immunohistochemistry (IHC) staining for critical markers like HNF1A and KRT81, a labor‐intensive manual staining process that introduces variability. Virtual staining methods offer promising alternatives by generating synthetic IHC images from routine hematoxylin and eosin (H&E) slides. However, most current approaches evaluate success by image quality measures rather than assessing diagnostically relevant features. Here, we introduce a novel cycleGAN framework utilizing a contrastive‐inspired approach trained on semipaired datasets derived from consecutive tissue sections. Our method significantly enhances PDAC subtyping accuracy based on synthetic IHC images generated from standard H&E inputs, improving the classification F1‐score from 0.66 to 0.77 for KRT81 and from 0.61 to 0.73 for HNF1A, compared with classification directly on H&E images. This approach also substantially outperforms baseline CycleGAN models. These results underscore the clinical potential of contrastive virtual staining to streamline PDAC diagnostics and improve their robustness. © 2025 The Author(s). *The Journal of Pathology* published by John Wiley & Sons Ltd on behalf of The Pathological Society of Great Britain and Ireland.

## Introduction

Subtyping of tumors is critical for clinical decision‐making and predicting patient outcomes. Traditionally, subtyping relies on pathological examination of tissue slides that are stained with various immunohistochemical (IHC) markers. For pancreatic ductal adenocarcinoma (PDAC), two IHC markers, hepatocyte nuclear factor‐1A (HNF1A) and cytokeratin‐81 (KRT81), have emerged as practical surrogates for defining relevant PDAC subgroups [1, 2, 3]. However, manual subtyping performed by pathologists is inherently subjective, labor‐intensive, and time‐consuming, posing challenges for routine clinical practice [2, 4]. Therefore, automatic computational methods for tumor subtyping have emerged as promising approaches to overcome limitations associated with manual pathological assessments [5, 6, 7, 8, 9]. Predominantly, these automated methods focus on within‐domain tasks, such as delineating tumor regions on baseline hematoxylin and eosin (H&E)‐stained slides or predicting specific tumor subtypes from IHC staining. Automated approaches for cross‐domain predictions, such as directly inferring IHC‐based tumor subtypes from routine H&E‐stained slides, still pose significant challenges due to the translation of features between domains [10]. Consequently, despite advances in computational pathology, physical staining continues to be indispensable for accurately revealing distinct cellular patterns required for robust tumor subtyping.

One promising approach to mitigate these issues is virtual staining, which narrows the gap between the broadly available H&E stain and the specific stains that are required for tumor subtyping, such as HNF1A and KRT81 [11, 12, 13, 14, 15, 16, 17]. These approaches generate synthetic special stainings, such as IHC, directly from standard H&E‐stained slides. Theoretically, this reduces the dependency on multiple physical stainings and accelerates clinical workflows. While many of these methods produce visually plausible images, they frequently fail to achieve a biologically meaningful and spatially precise mapping between corresponding tissue features of different staining modalities. One contributing factor is the limited availability of paired datasets that contain identical tissue sections stained with multiple modalities, which are well‐suited for training virtual staining algorithms, but are rarely available. Consequently, their outputs cannot support reliable clinical subtyping [12, 18].

In this study we directly address these limitations by introducing a novel virtual staining method capable of generating synthetic IHC images of PDAC from unpaired datasets. To overcome the challenges associated with unpaired data, we propose an innovative strategy that transforms commonly available unpaired datasets into semipaired datasets. This improves spatial and morphological correspondences crucial for effective model training. Building upon this enhanced semipaired dataset, we developed a CycleGAN training framework inspired by contrastive learning principles, specifically tailored to optimize virtual staining performance. We demonstrate that our method significantly enhances the accuracy of PDAC tumor subtype classification based solely on H&E input images. Specifically, employing synthetic IHC images generated through our approach increased the classification accuracy from baseline values of 0.66 and 0.61 for subtyping markers KRT81 and HNF1A (achieved using standard H&E images) to 0.77 and 0.73, respectively. Furthermore, our method substantially outperforms the baseline CycleGAN, which only transfers style between domains without leveraging semipaired correspondences. This demonstrates the effectiveness of our contrastive‐inspired CycleGAN strategy for robust and accurate virtual staining in PDAC subtyping.

## Materials and methods

### Ethics approval and consent to participate

This study was approved by the Institutional Review Board (IRB) of the Technical University of Munich. All samples were extracted from patients who gave broad consent to use excess tumor material and anonymized clinical data for scientific studies according to the regulations and ethical votes of the Tissue Bank of the University Hospital of the Technical University of Munich – MTBIO (ethical vote Nr. 1926/2007 and 126/2016S).

### Virtual staining for PDAC subtype prediction

Our work aimed to improve the prediction of two IHC‐based KRT81 and HNF1A subtypes of PDAC, based on baseline H&E stainings. In the following sections, we describe a sampling strategy that transforms an unpaired dataset into a semipaired dataset, facilitating more effective training of our virtual staining algorithm. Building on this, we present a contrastive virtual staining framework aimed at improving PDAC subtype prediction.

### Dataset

Our dataset consisted of tissue microarray (TMA) cores derived from pancreatic resection specimens. For each subject, two biopsy cores (0.2 cm diameter) were sampled. From each core, three consecutive slices were cut and stained with H&E, KRT81, and HNF1A. Consequently, a subject could have up to six tissue slices in the dataset (two biopsy cores, from which three slices for the three stainings were acquired). The sections were spatially organized within a TMA, where each core was uniquely indexed based on its position in the array. Up to 60 cores were combined on a single slide. This dataset is representative of images that are commonly available in clinical routine diagnostics.

The reference standard (KRT81‐positive, HNF1A‐positive, or double‐negative) was determined per slide by expert pathological examination. For each subtyping, the pathologist categorized staining intensity into negative, weak, medium, and, strong for the respective IHC slide. In combination with the percentage of stained tumor cells, final subtype classification (HNF1A‐positive/KRT81‐positive, or double‐negative) was determined, based on the physically stained IHC slide of a subject [1, 2]. Therefore, only physical KRT81‐ or HNF1A‐stained slides were examined to determine KRT81 or HNF1A positivity or negativity for a subject

The dataset initially comprised 149 subjects, although only TMA cores with a complete set of all three stainings were included in the final dataset. Cutting or preparation errors might have caused overlapping or separation artifacts in the tissue sections. While this did not pose a problem for diagnostic purposes, we excluded all affected sections from this core for our training, as typically two biopsies were sampled. Consequently, only one biopsy core per subject was used in such cases.

**Figure 1:**
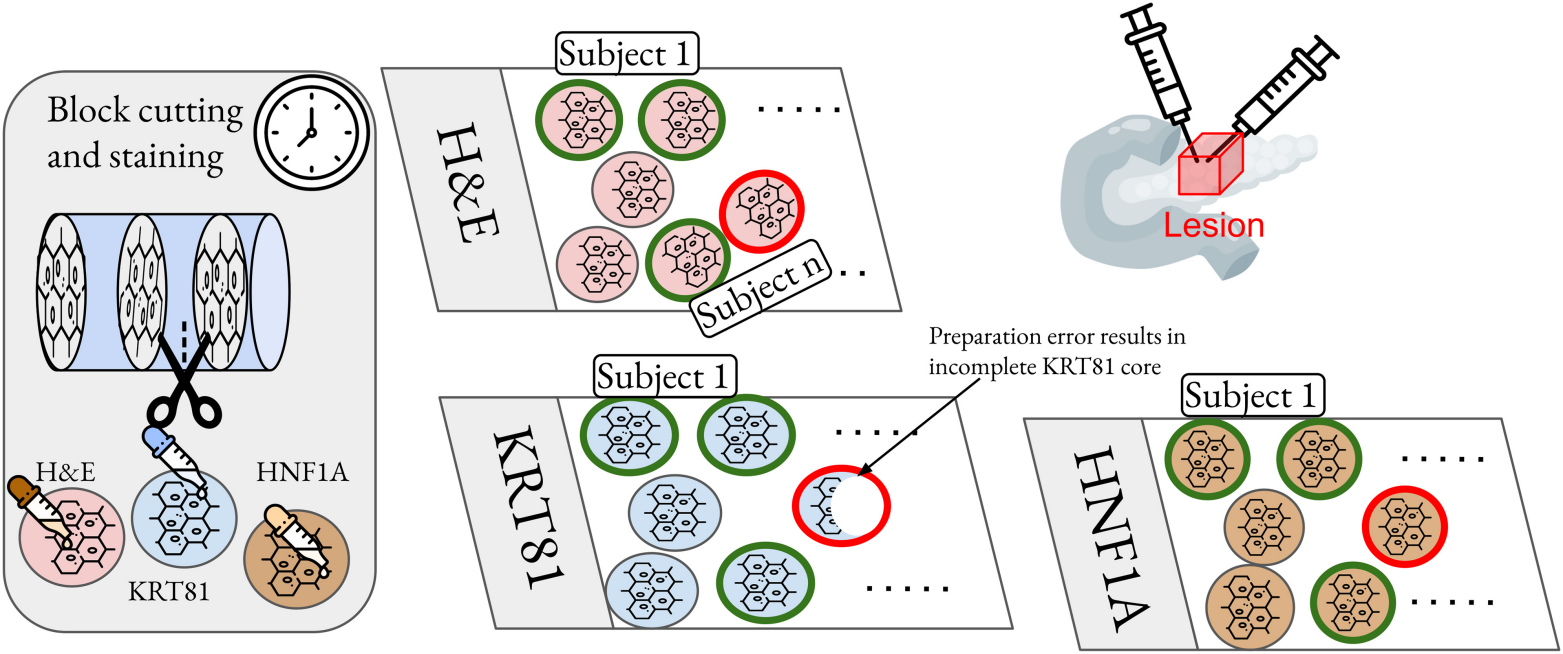
For each subject, usually two biopsy cores are sampled to definitely sample the lesion. From each of the two cores, one H&E, one KRT81, and one HNF1A staining are acquired on three consecutive slices from the biopsy core. Cases where one of the stained spots was corrupted by cutting artifacts were neglected in our dataset.

After filtering out incomplete cases, 140 subjects remained: 138 subjects with two cores (six slices) and two subjects with one core. Additionally, nine cases were excluded due to missing pathological ground truth. After data cleaning, the final dataset comprised 34 KRT81‐positive, 40 HNF1A‐positive, and 66 double‐negative subjects. An overview of the data preparation step is shown in Figure 1.

All samples used in this study were obtained from patients who underwent tumor resection at the Department of Surgery of the TUM School of Medicine and Health, Technical University of Munich. Samples were analyzed at the Institute of General and Surgical Pathology, Technical University of Munich between 2018 and 2019.

All IHC staining followed previously published protocols [2]. In brief, IHC staining was performed on a Ventana BenchMark XT (Ventana Medical Systems, Tucson, AZ, USA) after antigen retrieval. Primary antibodies rabbit polyclonal anti–HNF‐1A (Santa Cruz Biotechnology, Dallas, TX, USA, cat no. sc‐8,986; dilution 1:100), and mouse monoclonal anti‐Keratin 81 (Santa Cruz Biotechnology, cat no. sc‐100,929; dilution 1:500) were incubated for 2 h at room temperature. Detection was performed using the Dako Real Peroxidase Detection System Kit cat. No. K5007; Agilent, Santa Clara, CA, USA following the manufacturer's specifications, including the ready‐to‐use anti‐rabbit/mouse secondary antibody (cat no. K5003).

To assess reproducibility, two additional TMA blocks were generated as a validation cohort, following the same standard procedure as the main cohort. These consisted of tumor material from 20 additional patients with PDAC, resected between 2008 and 2016, which were not included in the main patient cohort.

### Semipaired dataset

Accurate subtyping of PDAC critically depends on both the staining intensity and the number of positively stained tumor cells. However, neither parameter can be directly inferred from H&E‐stained slides alone. Nevertheless, if the specific cells requiring staining are known, the number of positively stained cells per biopsy core can be automatically determined. The main challenge in PDAC subtyping from H&E images is therefore identifying which cells visible on H&E slides should exhibit IHC staining and at what intensity. This task is particularly difficult without paired datasets, in which H&E and the corresponding IHC slides are precisely matched.

As can be seen in the supplementary material, Figure S1, large offsets were present between consecutive slices. Landmarks across slides were often difficult to track, or even completely absent. The use of image registration for achieving pixel‐wise alignment was therefore unfeasible. Instead, we used rough landmark matching, similar to previous approaches [19, 20] to align morphologically comparable regions across slices. Since paired datasets must, by definition, contain the exact same tissue sections [21, 22, 23, 24], we refer to our dataset as a semipaired dataset. Here we aimed to generate weakly paired data consisting of H&E patches and corresponding IHC patches matched according to the staining intensity domains.

Staining intensity acted as a key criterion. We first identified regions of interest exhibiting distinct staining intensities on the IHC slides for two markers, KRT81 and HNF1A, which distinctly stain the cytoplasm and the cell nuclei brown, respectively, providing clear visual differentiation from the lighter, predominantly white, background. Within individual subjects, the staining intensity was generally homogeneous across the slide; multiple areas with differing intensities rarely occurred. Therefore, once a stained region on an IHC slide was identified, the staining intensity (weak or strong) could be directly inferred from the pathologist's ground truth annotations.

**Figure 2:**
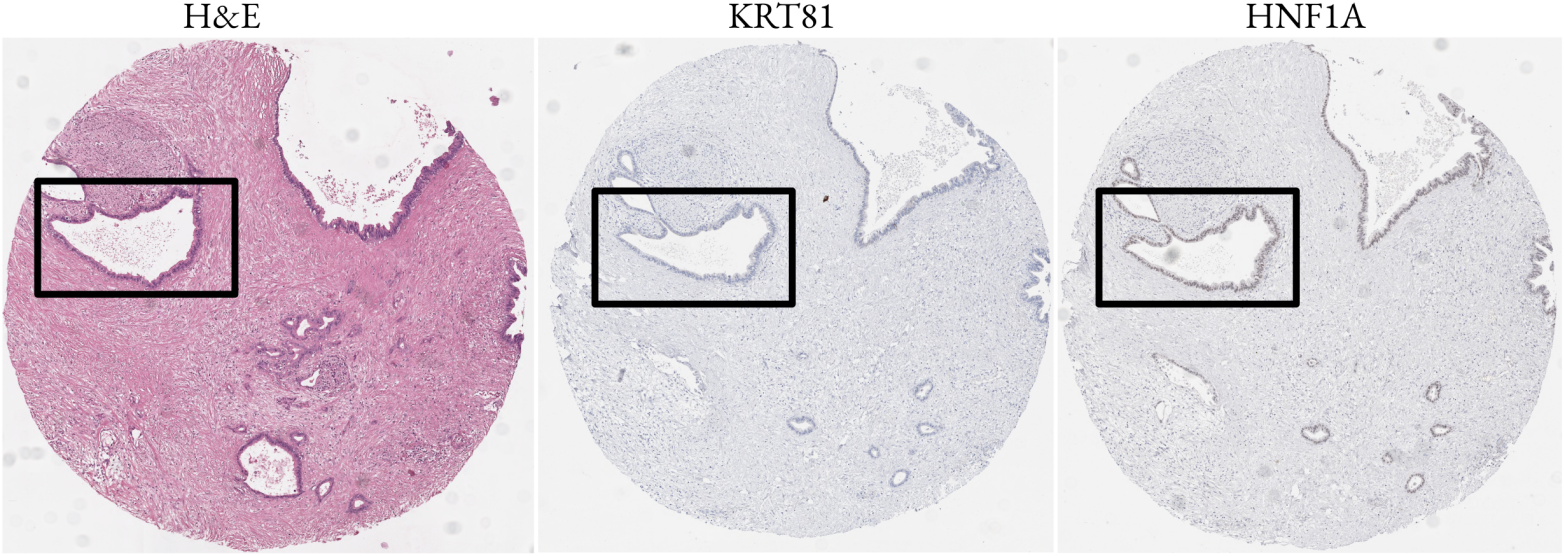
Three consecutive slices from one core shown next to each other. Despite consecutive slices, larger anatomical regions can be clearly identified. We used these anatomical landmarks to detect corresponding regions from which we sample patches.

While unpaired datasets do not correspond precisely, consecutive tissue sections are often sufficiently aligned to allow tracking of major morphological structures, such as tumor regions, across multiple sections. We leveraged this spatial proximity by manually detecting anatomical landmarks, such as prominent blood vessels or identifiable holes present on each core, to match regions between corresponding H&E and IHC slides. Figure 2 illustrates an example of how we matched corresponding sections across the three consecutive slices from one biopsy core.

For instance, after identifying a region with weak staining intensity in an IHC core stained for KRT81, we spatially mapped this region onto the corresponding H&E core using these predefined landmarks. Subsequently, patches were sampled from the matched regions on both the H&E and IHC slides. This process was repeated independently for each staining intensity level and separately for both KRT81 and HNF1A markers. As a result, we created pairs of H&E and corresponding IHC patches for each marker and staining intensity, effectively approximating how the H&E‐visible cells would appear had they been stained immunohistochemically.

### Domain aware virtual staining

**Figure 3:**
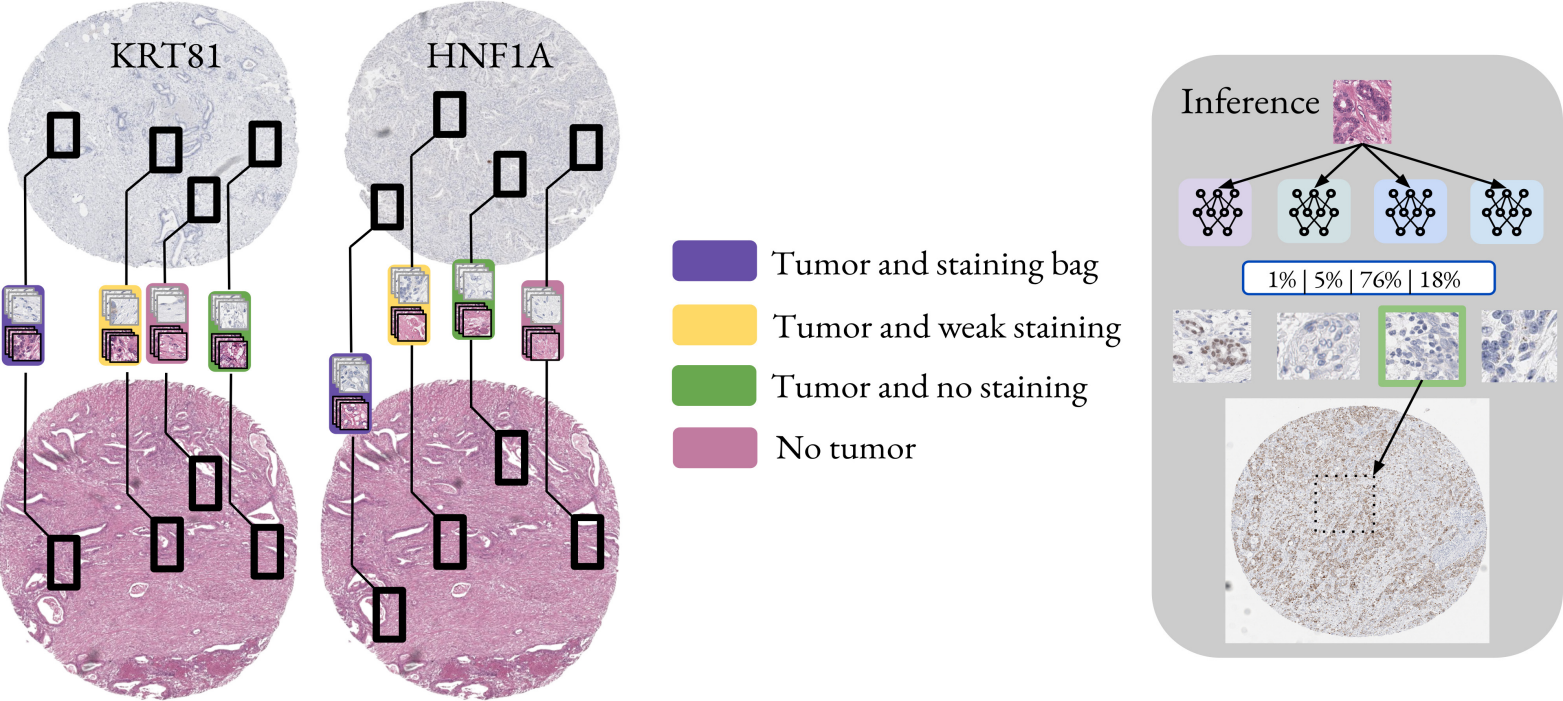
Visualization of the bag creation process. We correlated regions in both the H&E and the respective IHC images to each other. Since we correlated regions from one biopsy with each other, we show the same H&E image twice. From regions that we identified as correlating, we sampled patches. We only show the four bags for visualization purposes on cores from one subject. Typically, one subject exhibited for stained cells only one intensity, either strong or weak. The images that are sampled from one diagnostically relevant region are shown in the respective colorized box. During inference, we selected the GAN model with the highest confidence to generate the synthetic staining.

**Figure 4:**
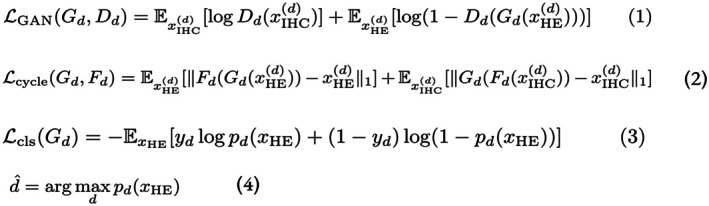
Combined presentation of all equations used.

With this special dataset, we next proceeded with the training of our stain transfer model. Semantic distortions are a known problem for CycleGAN models. Previous studies that applied CycleGAN models for virtual staining methods in digital pathology usually incorporate some sort of constraint during training to streamline the model's performance towards producing biologically correct synthetically stained images. Previous studies use various types of constraints for CycleGAN models. Most common are additional saliency constraints to reduce hallucination [25], or pathology consistency constraints that are enforced with a pathological representation network [15]. In contrast to previous work, we constrained our CycleGANs with a dataset‐derived supervision approach derived from our sampled dataset bags. Figures 3 and 4 illustrate this approach, as well as the supplementary material, Figure S1. Since we also introduced out‐of‐domain patches (*negative patches*) during training of our CycleGAN, we refer to our approach as contrastive CycleGAN. However, it is important to note that this is only a contrastive‐inspired approach and no real contrastive learning, which refers to a type of pretraining of encoder models used in previous works [26, 27].

### Subtype classification

In contrast to previous studies, our primary objective was not to generate the most visually realistic synthetic stains, but rather to develop an improved subtyping framework for identifying IHC subtypes based on H&E input images [16, 28]. To achieve this, we integrated a virtual staining algorithm with a dedicated classification approach, employing a Multiple‐Instance‐Learning (MIL)‐based framework, which is widely utilized in computational pathology [8, 9, 29]. In MIL approaches, a given WSI is split into patches. An encoder model transfers each input patch from the given WSI into a lower‐dimensional feature embedding. The aggregated low‐dimensional representation of a WSI is passed through a final classifier to obtain a classification result. Several specialized approaches emerged to tailor MIL frameworks specifically to pathological use cases. One customized MIL framework imitates the zooming of pathologists on slides and combines features of various resolution levels [26]. We also used this approach for PDAC classification and extracted features from multiple resolution levels to capture the staining intensity from the high‐resolution patch, as well as the amount of staining from the context patch.

### Experiments

In all our experiments, we performed five‐fold cross‐validation, partitioning the dataset on the subject level, and throughout our experiments, the subject distribution across folds remained the same. For both the training of the virtual staining method and the classification mode, we used the same train‐test splits to prevent leakage between train and test splits between the experiments. Strictly speaking, no subject that was used for training the virtual staining model was used in the test split of the classification model. To compare different approaches, we only considered the ultimate IHC tumor subtyping performance based on H&E‐input images. In our experiments, we evaluated different virtual staining algorithms to narrow the performance gap between subtype prediction directly on H&E slides and on IHC slides.

### Virtual staining with domain awareness

As an implementation of our contrastive GAN training, we used the pix2pix framework to facilitate unpaired image‐to‐image translation [30]. However, for both transformations that need to be learned (H&E to KRT81 and H&E to HNF1A), we trained four GAN models within the four classes ‘tumor with staining,’ ‘tumor with weak staining,’ ‘tumor without staining,’ and ‘non‐tumor without staining.’ We trained all GAN models with the same hyperparameters: 400 epochs, a VanillaGAN backbone, Adam optimizer, and a batch size of 4.

To address the issue of mosaic artifacts that often occurs on patch boundaries of virtual staining models, we trained with a patch size of 4,096 to have generally fewer patch transitions occurring in the final synthetic TMI core, which was 10,000 × 10,000 pixels.

To enhance domain awareness, we introduced H&E patches during training, as described in the Supplementary materials and methods. As an ablation study for our contrastive‐inspired GAN training, we also trained two baseline CycleGANs for the mapping of HE→KRT81 and one for the mapping of HE→HNF1A. These models are trained without any form of supervision and trained on 100,000 random patches from each of the domains without any alignment between the patches. We refer to this model as *CycleGAN*. The respective synthetic IHC slides (either synthetic KRT81 or synthetic HNF1A) that we generated, either with our proposed contrastive CycleGAN or with the baseline CycleGAN, were all created offline before any part of the subsequent classification framework was trained.

### Feature extraction

**Figure 5:**
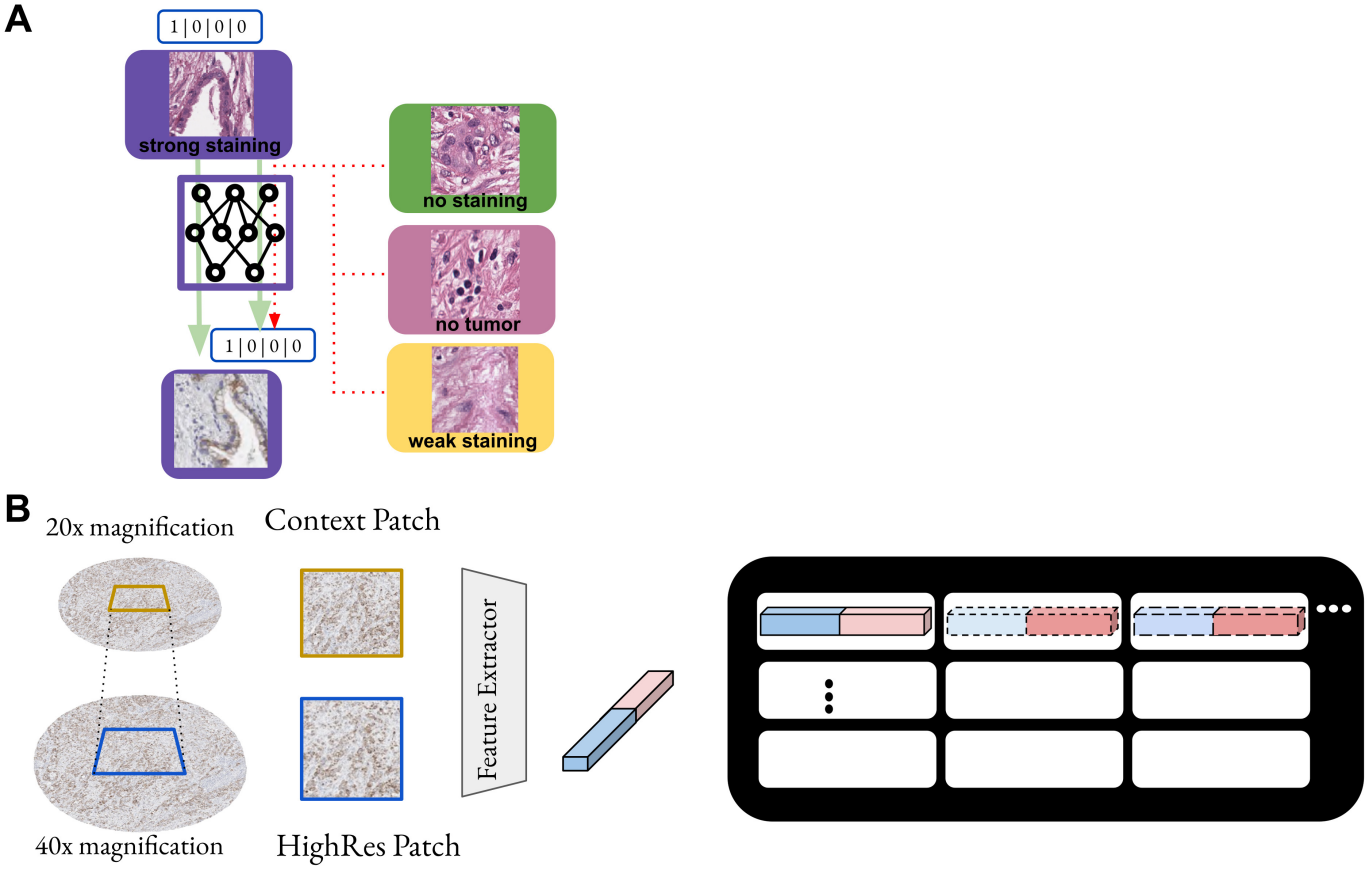
Schematic overview of our GAN training scheme. (A) Within each training bag, the discriminator learns to distinguish between in‐ and out‐of‐domain H&E input patches. (B) Visualization of the feature extraction process. We generated embeddings for the input images from two different resolutions. For presentation purposes, we only show one encoder here. After extracting the original features, we also performed augmentations, described in the previous section and computed the features again. We sampled the whole TMA core with this approach.

For feature extraction in our MIL framework for PDAC classification, we considered two encoders: a contrastive pretrained ResNet18 and the foundation model UNI2‐h [9]. The ResNet18 encoder was fine‐tuned for each input data domain, where we trained individual models for each input data, while we used the foundation model without any specific fine‐tuning. For pretraining of the ResNet18, we used the framework previously described [26], which is based on previous work by Chen *et al* [27]. Details on the contrastive pretraining for feature extraction can be found in the Supplementary materials and methods. In contrast to this, the UNI encoder was applied without modification or any domain‐specific finetuning. UNI is a CLIP Vision Transformer model from OpenAI and trained on a large‐scale collection of pathological slides. UNI has shown outstanding performance as a feature extractor for various computational tasks, like Multi‐label classification or disease subtyping. Given via the model architectures, we extracted 512 features per patch with the ResNet and 2,048 with UNI. For feature extraction, we sampled 224 × 224 shaped patches at 40× magnification and its corresponding section at 20× magnification, since we deployed the dual‐stream approach previously described [26]. For each patch, we therefore sampled 1,024 or 4,096 features. For one TMA core, this resulted in approximately 1,000–1,200 patches. Additionally, we applied augmentations during feature extraction besides plane feature extraction. Since we augmented the input patches and not the features, we also had to apply augmentations during feature extraction. Therefore, we computed features for the original patch, as well as for the patch when ColorJitter, Grayscale, GaussianBlurring, RandomHorizontalFlip, RandomVerticalFlip or GaussianNoise was applied. During MIL training, we then randomly selected one of the augmented feature sets. A schematic illustration on how each feature bag per TMA‐core was constructed is shown in Figure 5. We selected both models as feature extraction to also evaluate if large‐scale pretrained and highly compute‐intensive Vision Transformer models or lightweight domain‐specific feature extraction models perform better for PDAC classification. All patch‐sampling steps were performed using the Python library OpenSlide [31].

### Subtype classification

For the ultimate classification task, tumor subtyping into KRT81‐positive, HNF1A‐positive, or double‐negative, we used an MIL framework. Classification performance of the MIL framework was assessed using different types of input images, while all hyperparameters during subsequent MIL training were kept constant.

For the MIL‐framework, we used the DSMIL framework [26], implemented using pytorch [32]. Our model consisted of an instance classifier and a bag classifier within an MIL framework. Dropout regularization was applied at both the patch and node levels to improve generalization. During training, augmented embeddings of the patches were randomly selected from the bag features. The MIL network produced both instance‐ and bag‐level predictions, where the maximum instance response was combined with the bag prediction for loss computation. The final loss function was formulated as a weighted sum of bag‐level cross‐entropy loss and a scaled instance‐level loss, balancing global and local decision‐making. Experimentally, we determined the ideal weighting factor of the instance‐level loss as 0.2.

All DSMIL models were trained for 3,000 epochs, with a learning rate of 0.0002, a patch dropout probability of 0.1, and a node dropout probability of 0.3. As loss function, we employed the Binary Cross Entropy Loss and AdamW optimizer. To address class imbalances, we under‐sampled the negative classes.

To determine model performances, we determined the F1 score and ROC‐AUC curves. As a metric, we used the F1‐score to address the class imbalances within the KRT81 and HNF1A classes. The F1‐score represented the harmonic mean between precision and recall, and thus quantified the relation between true‐positives and false‐positives. In our experiments, we predicted both IHC subtypes independently from each other and did not predict the combined IHC subtype for the subjects. For example, we predicted KRT81 positive or negative and HNF1A positive or negative individually for each subject.

## Results

**Figure 6:**
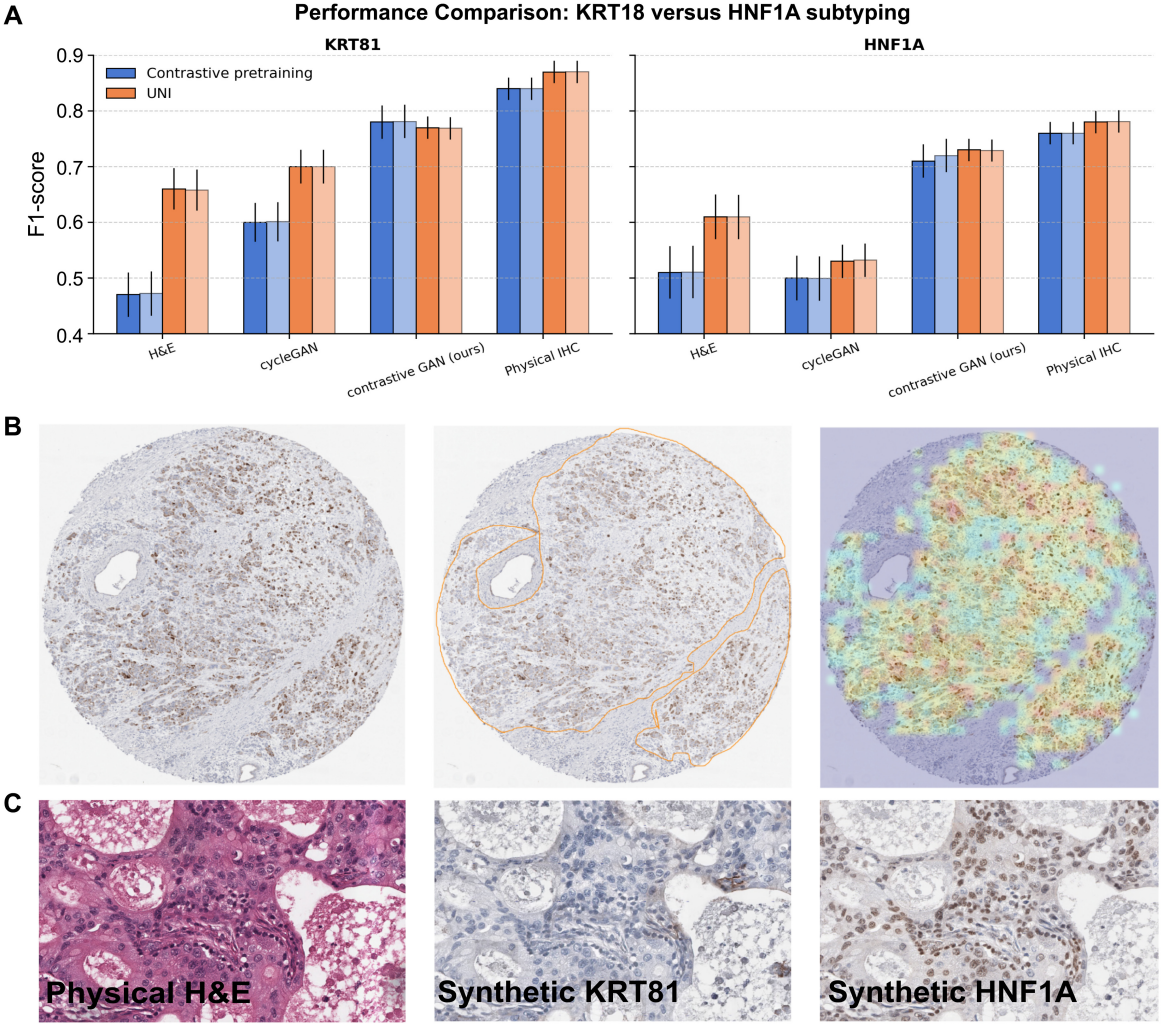
Results of the PDAC downstream analysis. (A) We report the performance of the MIL framework for various types of input images. H&E images, physical IHC images, and virtually generated IHC images, from H&E images. We compared two different feature extraction methods, marked in orange and blue. a domain‐specific finetuned ResNet18 and the foundational UNI model. The results on the additional test cohort are marked in light colors. (B) Left: Sample of one TMA spot for KRT81. Middle: Corresponding pathologist GT annotation for tumorous regions. Right: Tumor heatmap of the MIL framework for a synthetic IHC slide that was generated with the contrastive GAN model. (C) Left: One ground truth H&E‐stained image patch. Middle: The corresponding model generated KRT81 staining. Right: The corresponding model generated HNF1A staining.

For evaluation of subtyping performances based on H&E input images, we report the F1‐score of the MIL framework. For each input dataset, an MIL‐model was trained and accuracy assessed during five‐fold cross‐validation. All predictions were stacked from the respective folds on the validation set and calculated as a single final F1‐score; thus, we do not report any standard deviations. A bar plot for the different settings is shown in Figure 6A and the AUCs in the supplementary material, Figure S2 and Table S1.

The highest subtyping performance was achieved for each of the two stainings with the physically stained slides. For H&E input images, the UNI foundation model used as a feature extractor seemed to extract more relevant features for the classification than a fine‐tuned ResNet18 model. Additionally, our proposed training scheme outperformed the baseline CycleGAN setting without any domain knowledge for the KRT81, or the HNF1A subtyping task. While the baseline CycleGAN already partially narrowed the gap between H&E and physical staining for KRT81, for HNF1A, no meaningful mappings were learned with the baseline CycleGAN.

Directly linked to the downstream performance was the capability of the discriminator models to detect in‐ and out‐of‐training‐domain patches (Table 1). As our study was specifically tailored to the fully automated PDAC subtyping task, we did not consider any evaluations on the synthetic IHC slides besides downstream performance.

**Table 1:** Classification performance of discriminator models and below the MIL classification performance with confidence intervals.

Domains	Tumor (stained)	Weak staining	No staining	No tumor
KRT81 (test set)	0.73 (0.72)	0.69 (0.70)	0.61 (0.60)	0.79 (0.79)
HNF1A (test set)	0.69 (0.69)	0.68 (0.69)	0.64 (0.64)	0.7 (0.71)

Classification performance	H&E [Confidence Scores]	cycleGAN	contrastiveGAN (ours)	Physical IHC
KRT81 (contrastive)	0.470 [0.430, 0.51]	0.600 [0.565, 0.635]	0.780 [0.810, 0.750]	0.840 [0.820, 0.860]
KRT81 (test set, contrastive)	0.472 [0.431, 0.510]	0.601 [0.566, 0.636]	0.781 [0.811, 0.751]	0.840 [0.821, 0.861]
KRT81 (UNI)	0.660 [0.697, 0.623]	0.700 [0.730, 0.670]	0.770 [0.740, 0.80]	0.870 [0.850, 0.890]
KRT81 (test set, UNI)	0.658 [0.618, 0.698]	0.700 [0.730, 0.670]	0.768 [0.738, 0.798]	0.871 [0.891, 0.851]
HNF1A (contrastive)	0.510 [0.557, 0.463]	0.500 [0.540, 0.460]	0.710 [0.740, 0.680]	0.760 [0.780, 0.740]
HNF1A (test set, contrastive)	0.515 [0.562, 0.468]	0.499 [0.539, 0.459]	0.720 [0.750, 0.690]	0.780 [0.800, 0.760]
HNF1A (UNI)	0.610 [0.657, 0.563]	0.530 [0.570, 0.490]	0.730 [0.750, 0.710]	0.780 [0.800, 0.760]
HNF1A (test set, UNI)	0.609 [0.656, 0.562]	0.532 [0.562, 0.502]	0.728 [0.748, 0.708]	0.781 [0.801, 0.761]

To further assess the model's clinical utility, a board‐certified pathologist annotated tumor regions within the virtually stained slides and performed subtyping based on morphological features. We overlaid a heatmap generated by the MIL framework onto the annotated regions. This visualization allowed for a direct comparison between the MIL‐derived predictions and expert‐defined tumor boundaries, providing insights into the alignment of virtual staining with pathologist‐driven interpretations (Figure 6B). For easy access to the results by pathologists, we uploaded the results to a Kaapana‐Server [33, 34] and converted the heatmaps to DICOM ParametricMaps [35]. Figure 6C illustrates a high‐resolution detail. Here, the H&E patch is artificially transferred into the KRT81 and HNF1A domains with our approach.

## Discussion

In this study we propose a novel virtual staining approach for digital pathology using a CycleGAN to generate synthetic KRT81 and HNF1A stains from H&E slides. Our method addresses a central challenge in computational pathology: the scarcity of paired datasets and the absence of ground truth annotations for stains or morphological features. To overcome this, we adopted a contrastive pretraining strategy that captures both IHC signal and underlying biological structures essential for classification, an advancement over conventional CycleGANs, which typically prioritize style transfer without incorporating biological context. A key strength of our approach is the integration of domain knowledge during training. We utilized diagnostically relevant regions from semipaired datasets, aligning H&E and IHC patches from adjacent biopsy sections, thereby improving domain adaptation. Our results show that the trained CycleGAN effectively generates accurate virtual stains for both KRT81 and HNF1A, with promising clinical applicability. Heatmaps from the MIL framework further enhance interpretability by highlighting areas where model predictions align with expert judgment. In contrast, unsupervised baseline cycleGANs trained on unpaired datasets failed to produce biologically meaningful synthetic patches. Rather than performing classification directly on cycleGAN intermediate latents, we opted to synthesize virtual IHC slides, which enables the application of established MIL pipelines without modification. This also reduces the need for physical IHC staining, a costly and labor‐intensive process, while improving transparency in diagnostics. Synthetic slides provide visual interpretability, allowing clinicians to assess discrepancies between model predictions and expert opinion.

We acknowledge that the reliance on a semipaired data generation approach, while demonstrated to be effective for virtual staining, inherently presents limitations regarding ground‐truth alignment and model supervision when compared to registration‐based strategies employing serial stainings of the same tissue section. The latter, by leveraging the anatomical continuity within a single tissue section and precise image registration techniques, generally offers a superior level of spatial correspondence between H&E images and their corresponding IHC ground truth.

Despite careful manual annotation and quality control, subtle biological variations between adjacent tissue sections, as well as potential tissue distortions during processing and mounting, can introduce minor spatial discrepancies. Thus, features learned by the model from the H&E image may not perfectly correspond pixel‐for‐pixel to the exact protein expression on the IHC ground‐truth. Consequently, this can introduce a degree of noise into the model supervision signal, potentially leading to less precise learning of molecular features and impacting the overall robustness and fine‐grained accuracy of the synthetic staining.

Our initial attempts to achieve such precise registration for our dataset encountered excessive spatial offsets, rendering direct pixel‐level alignment unfeasible. Consequently, we adopted this semipaired approach, which relies on soft pixel correspondences. It must be noted that this approach is generally inferior to approaches with a 1:1 mapping of the datasets. Furthermore, we observed performance differences between biomarkers, with KRT81 yielding more robust virtual staining than HNF1A. This likely reflects the underlying biological variability, as KRT81, a cytoskeletal protein, typically induces more reproducible and distinct morphological changes than the nuclear transcription factor HNF1A. This highlights a broader limitation in virtual staining, where the reliability and interpretability of the synthetic output are intrinsically impacted by the marker‐specific morphology and the precision of the ground truth used for training.

In conclusion, our CycleGAN‐based virtual staining approach, informed by domain knowledge and validated by experts, shows strong potential to improve digital pathology workflows. By enabling accurate stain generation from H&E slides, it offers gains in diagnostic efficiency, cost reduction, and interpretability, with the potential to support clinical decision‐making and improve patient outcomes.

## Author contributions statement

MF, AM, MG, RB, PN and KM‐H performed the study concept. MF performed the experiments. AM, KS, LF, JTS and RB provided acquisition and interpretation of the data. PS, HG and LF provided infrastructure components. All authors contributed methodological and statistical analyses, read, and approved the final article.

## Supporting information


**Supplementary materials** **and methods**
**Figure S1**. Two TMA‐cores of one subject
**Figure S2**. ROC‐AUC curves of the classification model
**Table S1**. AUC scores for the classification task

## Data Availability

Data can be made available upon reasonable request to the corresponding author. Code availability: Our code will be publicly available at this repository: https://github.com/MIC-DKFZ/ContrastiveVirtualStaining
