# Supplementary material for: Contrastive virtual staining enhances deep learning‐based PDAC subtyping from H&E‐stained tissue cores
Source: J Pathol. 2025 Nov 4;268(1):89–98. doi: 10.1002/path.6491 (PMC12699241; doi:10.1002/path.6491)
Supplement: Supplementary file 1 — Supplementary materials and methods Figure S1. Two TMA‐cores of one subject Figure S2. ROC‐AUC curves of the classification model Table S1. AUC scores for the classification task [file PATH-268-89-s001.docx]

**Contrastive virtual staining enhances deep learning-based PDAC subtyping from H&E-stained tissue cores**

M Fischer, A Muckenhuber *et al. J Pathol* <https://doi.org/10.1002/path.6491>

**Supplementary materials and methods**

**Supplementary Figures S1–S2**

**Supplementary Tables S1**

**Supplementary materials and methods**

As described in the main article, we have sampled data pairs from four diagnostically relevant regions: *tumor with staining, tumor with weak staining, tumor without staining, and non-tumor without staining* that contain H&E and IHC patches. We have individual bags for KRT81 and HNF1A. For each staining pair (e.g., H&E and KRT81 patches with weak tumor staining), we trained a separate cycleGAN model, specifically tailored to the respective staining domain, where each model $G_{d} : X_{HE}^{(d)} \to X_{IHC}^{(d)}$ learns a bidirectional mapping. The adversarial and cycle consistency losses are defined in equations 1 and 2, with *D_d_* as the discriminator and *F_d_* as the inverse mapping. For presentation purposes, we combine the individual models $G_{d} : X_{HE}^{(d)} \to X_{KRT81}^{(d)} and G_{d} : X_{HE}^{(d)} \to X_{HNF1a}^{(d)}$ as the *IHC* model, while for each diagnostic region and for each staining, we trained individual cycleGAN models. With four diagnostic regions and two stainings, we trained 8 cycleGAN models.

Unlike conventional CycleGAN applications where the domain definitions are coarse (e.g., H&E *versus* IHC globally), our setup enforces a much finer-grained domain structure. This ensures that subtle morphological differences between tumor regions with varying staining intensities are explicitly modeled. By training dedicated models for each diagnostic region, we aimed to capture region-specific staining phenotypes, which is especially relevant for weak or heterogeneous biomarker expression. Training independent generators for each diagnostic region is mathematically equivalent to partitioning the data distribution into four conditional domains $p(x^{HE} | d)$. This allows each generator to approximate a simpler conditional mapping $p(x^{IHC} | x^{HE} ,d)$ rather than the much harder unconditional mapping across all domains. In practice, this factorization reduces mode collapse and yields more stable convergence of the adversarial loss.

To select the correct CycleGAN model from each of the four domains during inference, we introduced HE patches from other domains (but not from other stainings) $X_{HE}^{(d')} (d' \neq d)$ during training of each cycleGAN, forcing each *G_d_* to learn domain specificity. We incorporated an auxiliary classification objective, that is described in equation 3. where *y_d_* is the binary domain label and *p_d_(x_HE_)* represents the model's confidence that *x_HE_* belongs to domain *d*. This can also be translated to an additional classification task that is trained on top of the CycleGAN model. At inference, given an unknown *x_HE_*, we computed domain probabilities *p_d_(x_HE_)* for all four trained models and selected the model with the highest confidence, as described in equation 4.

This classification-enhanced training scheme effectively transforms the discriminator into a multitask network: besides distinguishing real *versus* fake patches, it also predicts domain identity. This encourages the generator to not only learn stain translation, but also to preserve domain-discriminative features, improving robustness when applied to ambiguous or heterogeneous input patches.

Domain probabilities are provided as additional output of the discriminator. For updating the Generator, we used the discriminator signal for the *in-domain-patches* and the *out-of-domain-patches* weighting factor. To enhance domain awareness, we introduced H&E patches from alternative diagnostic categories into each model's training set with a probability of *p* = 0.3. For in-domain patches (e.g., a tumor with staining HE patch trained in the corresponding model), we set alpha, the weighting factor of our domain classification loss to alpha = 1, while for out-of-domain patches, we reduced the weight to alpha = 0.1 to prevent overfitting and encourage generalization across staining conditions. Empirically, this strategy allowed the model to learn discriminative cues specific to the diagnostic category (e.g., morphology of tumor regions with weak staining) while avoiding mode collapse towards a generic translation. The probabilistic injection of out-of-domain patches at *p* = 0.3 provided an effective trade-off: enough exposure to alternative morphologies to promote generalization, without overwhelming the domain-specific learning signal. Intuitively, this weighting creates an expected loss of roughly $0.7 \cdot\mathcal{L}_{in} + 0.3 \cdot\mathcal{L}_{out}$. Therefore, in-domain examples dominate learning, but out-of-domain examples act as a small regularizer, ensuring the generator does not overfit to narrow morphological cues. This probabilistic exposure functions like a soft regularization scheme, stabilizing training while maintaining domain specificity.

**Feature extraction**

During contrastive pretraining, we trained the model with the following augmentations: ColorJitter (*p* = 0.8), RandomResizedCrop (size = 224), RandomHorizontalFlip, RandomAutocontrast, RandomGrayscale (*p* = 0.2), GaussianBlur. We set the ColorJitter parameters to (0.1, 0.8, 0.8, 0.3) for H&E images and (0.8, 0.8, 0.8, 0.2) for IHC to order to preserve perceptual information in the generally brighter IHC images. All contrastive pretraining was performed with a batch size of 100 for 100 epochs, using a weight decay of 10^−6^ and a contrastive loss based on a cosine similarity with a temperature scaling factor of 0.5.

**A1: Semi-paired dataset**


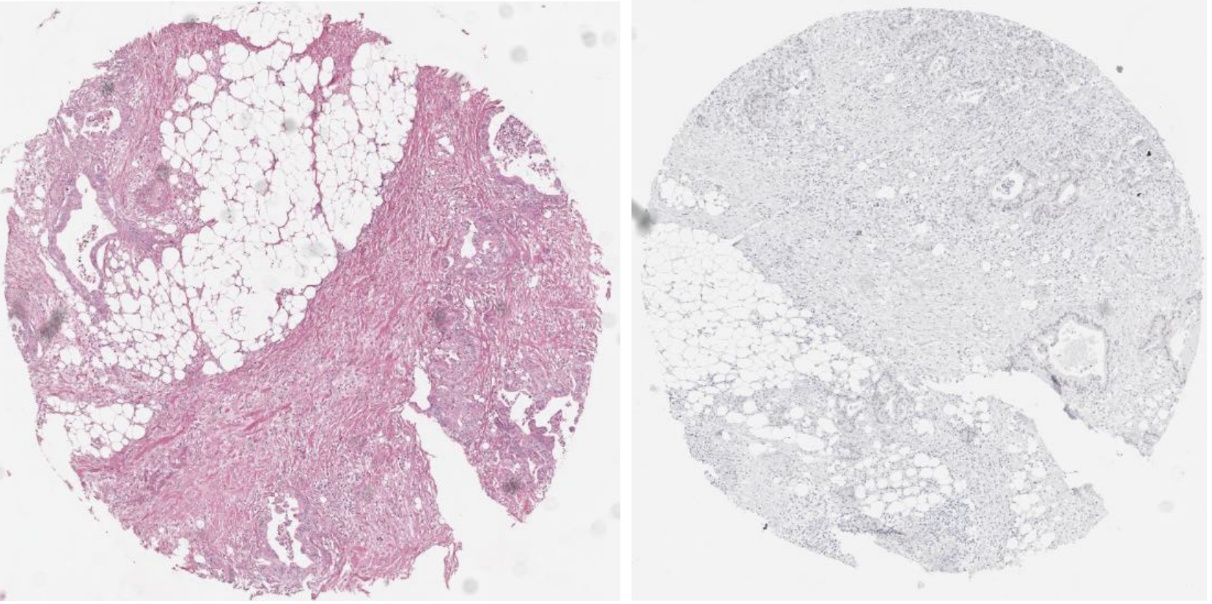


**Figure S1. Two TMA-cores of one subject.** The cores are from consecutive slices and a high morphological discrepancy can be observed. Using linear registration methods to achieve cellular alignment is thus not feasible.

**
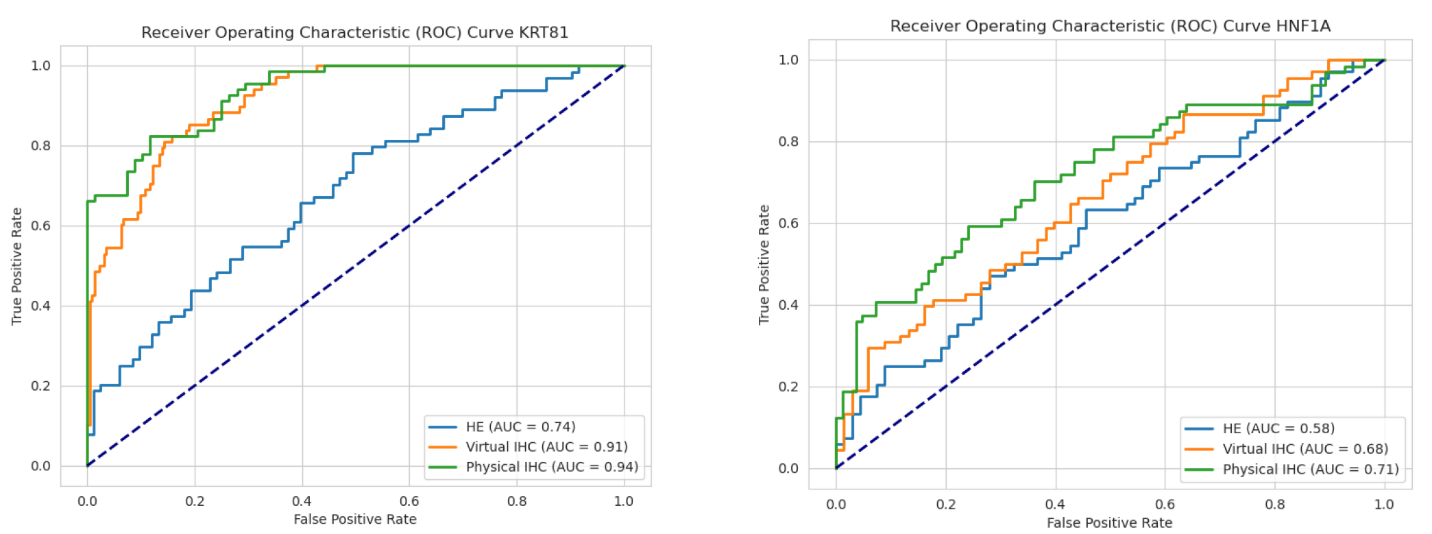
**

**Figure S2. ROC-AUC curves of the classification model.** Virtual IHC were generated using our contrastive GAN method.

**Table S1. AUC scores for the classification task.** Note, for concise evaluation, the F1-scores from the main article should be considered.

| **AUC** | **H&E** | **Cycle GAN** | **Contrastive GAN (ours)** | **Physical IHC** |
| --- | --- | --- | --- | --- |
|  | Contrastive pretraining/UNI | | | |
| KRT81 | 0.74/0.86 | 0.84/0.88 | 0.91/0.93 | 0.93/0.95 |
| HNF1A | 0.58/0.68 | 0.61/0.79 | 0.68/0.79 | 0.71/0.89 |
